# Health Care Professionals’ Experiences and Perspectives on Using Telehealth for Home-Based Palliative Care: Protocol for a Scoping Review

**DOI:** 10.2196/33305

**Published:** 2021-10-29

**Authors:** Elias David Lundereng, Andrea Aparecida Goncalves Nes, Heidi Holmen, Anette Winger, Hilde Thygesen, Nina Jøranson, Chrstine Råheim Borge, Weiqin Chen, Olav Dajani, Kari L Mariussen, Simen A Steindal

**Affiliations:** 1 Section for Palliative Treatment Department of Oncology Oslo University Hospital Oslo Norway; 2 Lovisenberg Diaconal University College Oslo Norway; 3 Faculty of Health Sciences Department of Nursing and Health Promotion Oslo Metropolitan University Oslo Norway; 4 Faculty of Health Studies VID Specialized University Oslo Norway; 5 Department of Occupational Therapy, Prosthetics and Orthotics Oslo Metropolitan University Oslo Norway; 6 Lovisenberg Diaconal Hospital Oslo Norway; 7 Department of Interdisciplinary Health Sciences University of Oslo Oslo Norway; 8 Department of Computer Science Oslo Metropolitan University Oslo Norway; 9 Section for Gastrointestinal Oncology Department of Oncology Oslo University Hospital Oslo Norway

**Keywords:** health care technology, home care services, palliative care, review, telehealth, telemedicine

## Abstract

**Background:**

Telehealth seems feasible for use in home-based palliative care. However, acceptance among health care professionals (HCPs) is essential for the successful delivery of telehealth in practice. No scoping review has mapped the experiences and perspectives of HCPs on the use of telehealth for home-based palliative care.

**Objective:**

The aim of this review is to systematically map published studies on HCPs’ experiences and perspectives on the use of telehealth in home-based palliative care.

**Methods:**

The proposed scoping review will employ the methodology of Arksey and O’Malley. This protocol is guided by the Preferred Reporting Items for Systematic Review and Meta-Analysis Protocol (PRISMA-P). A systematic search will be performed in MEDLINE, PsycINFO, EMBASE, CINAHL, Allied and Complementary Medicine (AMED), and Web of Science for studies published between January 2000 and July 5, 2021. We will also hand search the reference lists of included papers to identify additional studies of relevance. The search will be updated in 2022. Pairs of authors will independently assess the eligibility of studies and extract data. The first 2 stages of thematic synthesis will be used to thematically organize the data. Because the scoping review methodology consists of reviewing and collecting data from publicly available materials, this study does not require ethics approval.

**Results:**

The database searches; testing of eligibility criteria; and screening of titles, abstracts, and full-text papers will be performed by fall 2021. The results from this scoping review will be presented as a descriptive summary of the results from all included papers, and will be inductively organized into descriptive themes. A frequency table illustrating which papers were included in which descriptive themes will be made. Results are anticipated by the fall of 2022.

**Conclusions:**

A mapping of studies could identify research gaps regarding HCPs’ experiences and perspectives on the use of telehealth in home-based palliative care and may determine the value and feasibility of conducting a full systematic review.

**International Registered Report Identifier (IRRID):**

PRR1-10.2196/33305

## Introduction

More people will require palliative care in the future due to the growing number of older people and the increasing prevalence of chronic illnesses [1,2]. Palliative care is an approach that aims to improve the quality of life of patients and their families facing life-threatening illness. Palliative care is applicable early in the course of illness, in conjunction with treatments that intend to prolong life [3]. Palliative care is relevant for various diseases and conditions such as cancer, dementia, chronic lung diseases, and heart diseases [4]. Most patients receiving palliative care prefer to be cared for and spend as much time as possible in their own homes [5,6]. Home-based palliative care is associated with a reduction in symptom burden and increased patient and caregiver satisfaction [7]. A key goal in palliative care is to enable patients to spend more time at home by providing access to coordinated, continuous, and specialized palliative care at home [8]. However, many patients experience that the palliative care trajectory is unpredictable, and complaints about uncoordinated care, unmet palliative care needs at home, lack of regular communication with both health care professionals (HCPs) and between specialist and home care professionals are common [9,10].

The recent and ongoing COVID-19 pandemic presents additional challenges for HCPs in providing home-based palliative care. Physical distancing requirements, lockdowns, and lack of personal protective equipment may limit the access to home-based palliative care. Subsequently, increased isolation and suffering may increase the care burden on the families and caregivers [11].

Telehealth is increasingly used in home care and is defined “as the provision of healthcare remotely by means of a variety of telecommunication tools” [12]. A scoping review [13] suggests that telehealth is feasible for use in home-based palliative care as it improves access to palliative care at home, promotes self-monitoring, and enhances patients’ feelings of security and safety [13]. There is a significant increase in health care costs in the final years of life [14], and it is expected that telehealth solutions may contribute to more efficient use of resources in palliative care by preventing and reducing hospital admissions, emergency department attendances, and deaths in hospitals [15-17]. It may also enhance collaboration between different health care services by improving the information flow [17,18]. Recent policy changes during the COVID-19 pandemic have reduced barriers to implement telehealth services and have promoted the use of telehealth in palliative care as a way to improve communication between isolated patients and their families, and between patients and HCPs [19,20].

Although the possibilities within telehealth appear promising in facilitating high-quality care for various conditions, many HCPs feel that technology is inappropriate for the palliative care population due to age, burden of illness, and rapidity of deterioration [16]. The most common fear of technology is that machines will replace all human contact [21]. Previous studies have shown that HCPs may characterize palliative care as high touch rather than high tech and concerns about telehealth being burdensome for the patients may limit their interest in implementing and applying telehealth solutions [22,23]. Further, reduced face-to-face contact with patients and lack of acceptance of this way of working among HCPs seem to be barriers in implementing telehealth in palliative care [24].

Previous literature reviews regarding the use of telehealth in palliative care have primarily examined patient or caregiver outcomes and experiences [13,17,23,25] or have focused on elderly patients, patients with chronic conditions [26,27], or children in need of palliative care [28,29].

Bienfait et al [22] published a systematic review regarding HCPs’ perceptions of using telehealth for monitoring patients with chronic disease and how those findings could transpose to palliative care. Another systematic review examined the use of video consultations in general and specialized palliative care to various patient groups from the perspectives of patients, caregivers, and HCPs [30]. They found that HCPs were positive toward the use of video consultations in palliative care, but expressed concerns regarding technical challenges, increased workload, and the required additional training in how to conduct video consultations. A systematic review [31] examined the use of technology for communication in palliative care from the perspectives of HCPs, patients, and caregivers. They found that the use of technology for communication efficiency resulted in improved quality of care and communication, and reduction of documentation efforts and overall health care costs. However, they did not report nor investigated HCPs’ experiences or perspectives of utilizing technology apps in home-based palliative care [31].

With the emergence of new research regarding the use of telehealth in palliative care, there is a need for a comprehensive review of the existing literature on how HCPs experience and perceive the use of telehealth to deliver home-based palliative care. Identifying facilitators and barriers for implementation of telehealth among HCPs is important, as acceptance of this way of working is essential to the successful delivery of telehealth in practice [32]. In line with the recommendations of Arksey and O’Malley [33], we have chosen to conduct a scoping review, as this allows the inclusion of studies with different study designs and is suitable to describe findings and range of studies within the field of telehealth. A mapping of studies could also identify research gaps regarding telehealth in palliative care and may determine the value and feasibility of conducting a full systematic review. Further, a scoping review may also be suitable to more accurately define a research question for a systematic review [33] and is also suitable to bringing together literature in disciplines with emerging evidence [34]. To the best of our knowledge, no scoping review has targeted the experiences and perspectives of HCPs on the use of telehealth for home-based palliative care. Consequently, the aim of this scoping review is a systematic mapping of published studies on the use of telehealth in home-based palliative care with focus on experiences and perspectives of HCPs. The research question is: What is known from published studies about HCPs’ experiences and perspectives on using telehealth for home-based palliative care?

## Methods

### Overview

Scoping reviews begin with the development of a protocol that aims to predefine the objectives and methods of the scoping review and detail the proposed plan. Because of the more iterative nature of a scoping review in contrast to a systematic review, some deviations from the protocol may be necessary [34]. This scoping review will employ the methodology of Arksey and O’Malley [33], which consist of the following stages: (1) identifying the research question; (2) identifying relevant studies; (3) selecting studies; (4) charting the data; and (5) collating, summarizing, and reporting the results. The reporting of this protocol is guided by the Preferred Reporting Items for Systematic review and Meta-Analysis Protocols (PRISMA-P) [35], while the reporting of the upcoming scoping review will be guided by the PRISMA extension for scoping reviews (PRISMA-ScR) [36].

### Eligibility Criteria

The inclusion and exclusion criteria are shown in Table 1. The first and last author will independently test the inclusion and exclusion criteria on the same 5% of the retrieved studies to assess the robustness of the criteria in capturing relevant publications. The inclusion and exclusion criteria may be revised based on conflicts during or after this initial testing. Once the final set of criteria is agreed upon, the entire group of researchers will screen the remaining titles and abstracts. The language criteria are based on authors’ fluency in the included languages.

**Table 1:** Inclusion and exclusion criteria.

Criterion	Inclusion	Exclusion
Types of studies	Qualitative, quantitative, or mixed methods studies.	Any type of review, case report, letter, book chapter, guideline, comment, discussion, editorial, conference abstract, study protocol, master’s thesis, or PhD thesis.
Period	January 1, 2000, until the updated search.	Before January 1, 2000, and after the updated search.
Language	English, Chinese, Portuguese, Spanish, Norwegian, Swedish, or Danish.	All other languages.
Type of participants	Papers including HCPs^a^ using telehealth with patients in home-based palliative care.	Papers including HCPs using telehealth with patients outside of a palliative care environment, those that only tend to family caregivers, or studies that do not present data from the perspective of HCPs.
Phenomenon of interest	HCPs’ experiences of and perspectives on the use of telehealth in home-based palliative care.	HCPs’ experiences of and perspectives on the use of telehealth at home without interaction with the patient, or experience of use of telehealth in hospital, nursing home, or hospice. Telehealth including only telephone follow-up.

^a^HCP: health care professional.

### Information Sources

We aim to perform a systematic search in the following electronic databases: MEDLINE, PsycINFO, EMBASE, CINAHL, Allied and Complementary Medicine (AMED), and Web of Science.

### Search Strategy

The search strategy in MEDLINE (Multimedia Appendix 1) will be built by an experienced research librarian (KM) and 2 of the other authors (EL and SS) using MeSH terms and text words. The search will consist of 3 elements: (1) palliative care, (2) telehealth, and (3) home setting. The search strategy will be piloted to validate appropriateness of text words and MeSH terms, and will be peer reviewed by a second experienced research librarian (MAØ), using the Peer Review of Electronic Search Strategies checklist [37]. The search strategy will then be adopted for each database. The database searches will be updated 2 months prior to publication. We will also hand search the reference lists of included papers to identify additional studies of relevance.

### Data Management

The research librarian will upload the publications identified in the searches to EndNote for removal of duplicates and transfer the publications into the web application Covidence [38] to facilitate independent selection of eligible publications, as well as storage.

### Selection Process

Pairs of authors will independently screen titles, abstracts, and full-text papers to determine their eligibility. EL and SS will solve potential conflicts among the pairs based on consensus. The study selection process will be reported using the PRISMA flowchart [39], alongside the reasons for exclusion of full-text publications.

### Data Collection Process

A standardized data charting form in Covidence will be developed and used to chart relevant data from the included papers. The following data may be included: authors, publication year, country, aim, sample, telehealth solution, design, and results related to the research question. The data charting form will be reviewed by the entire research team and pilot tested by the first and last author on 5 studies to ensure that the form is capturing the information accurately. More studies may be piloted based on the number of included studies. Based on these experiences, the data charting form may be revised. Pairs of authors (EL/SS, AN/HH, WC/AW, CB/EL, NJ/HT, and SS/OD) will conduct the data charting. One author will extract data, while the other author will check accuracy. Any discrepancies will be further discussed among the pairs of authors and agreement will be based on consensus or the involvement of the first and last author.

### Risk of Bias and Quality Appraisal

A key difference between scoping reviews and systematic reviews is that the former is not intended to be used to critically appraise or assess the risk of bias of a cumulative body of evidence. Generally, scoping reviews aim to provide an overview of existing literature regardless of methodological rigor or risk of bias [36]. Therefore, the included sources of evidence in this review will not be assessed for risk of bias or methodological quality. This is in line with the framework of Arksey and O’Malley [33] and Tricco et al [36].

### Data Synthesis

A scoping review seeks to provide an overview of the data rather than synthesize the evidence like that in a systematic review. However, a scoping review still needs an analytic framework to present a narrative account of the data [33]. We will use the first 2 stages of thematic synthesis [40] to inductively organize our data. In stage 1 of the thematic synthesis, the data from the result section of the included studies will be read multiple times and line-by-line coding will be applied to identify patterns, similarities, and differences in the experiences and perspectives of HCPs on the use of various technological solutions in home-based palliative care. In stage 2, the codes will be compared for similarities and differences and organized into descriptive themes with low degree of abstraction and interpretation. The first, second, third, and last author will organize the data. The final descriptive themes will be determined by the authors through discussion and consensus among all the authors. The qualitative data analysis software NVivo (QSR International) [41] will be used to organize the data. The codes and the descriptive themes will be discussed with all members of the research team who have diverse research and clinical expertise regarding telehealth, palliative care, and chronic illness. This could enhance the trustworthiness of the results.

## Results

In a scoping review, the results may be presented in a logical, diagrammatic, or tabular form, or in a descriptive format that aligns with the objectives of the review [34]. The results from this scoping review will be presented as a descriptive summary of the results from all included papers, and will be inductively organized into descriptive themes. A frequency table illustrating which papers were included in which descriptive themes will be made. A figure illustrating the hierarchical coding tree may also be developed to further illustrate the results [39].

## Discussion

We introduced the rationale and design of a scoping review to answer our research question: “What is known from published studies about HCPs’ experiences and perspectives on using telehealth for home-based palliative care?”. Research indicates that telehealth is feasible for use in palliative care [13], may increase patient and caregiver satisfaction, and may contribute to increased resource efficiency in home-based palliative care [15-17]. However, research also indicates that HCPs may be a key barrier in implementing technology, and that acceptance of this way of working among HCPs is vital for the successful implementation of telehealth in practice [32]. This scoping review will be important to provide an overview on published studies on HCPs’ experiences and perspectives on the use of telehealth in home-based palliative care. This review may identify gaps in the existing literature and determine whether a full systematic review is feasible. Furthermore, this review could provide a deeper understanding of HCPs’ perspectives and experience in telehealth in home-based palliative care. This may aid policy makers and telehealth developers in designing user-centric, demand-driven telehealth solutions. Because the scoping review methodology consists of reviewing and collecting data from publicly available materials, this study does not require ethics approval.

## Appendix

Multimedia Appendix 1 Medline search strategy.

## Abbreviations

HCP: health care professional
PRISMA-P: Preferred Reporting Items for Systematic Review and Meta-Analysis Protocol
PRISMA-ScR: PRISMA extension for scoping reviews
