# Supplementary material for: Health Care Professionals’ Experiences and Perspectives on Using Telehealth for Home-Based Palliative Care: Protocol for a Scoping Review
Source: JMIR Res Protoc. 2021 Oct 29;10(10):e33305. doi: 10.2196/33305 (PMC8590185; doi:10.2196/33305)
Supplement: Multimedia Appendix 1 [file resprot_v10i10e33305_app1.pdf]

Multimedia Appendix 1: Search strategy MEDLINE

Database(s): **Ovid MEDLINE(R) ALL** 1946 to June 30, 2021

Search Strategy:

#	Searches	Results
1	Palliative Care/ or "Hospice and Palliative Care Nursing"/ or exp Terminal Care/ or Palliative Medicine/ or exp Advance Care Planning/ or Terminally ill/	106824
2	(palliative or palliate* or palliating).tw,kf.	71977
3	((terminal* or "end stage*" or endstage* or "advanced stage*" or "late stage*") adj3 (disease* or ill* or care* or caring or treatment* or period* or nurs* or patient*)).tw,kf.	101882
4	(eol or "end of life").tw,kf.	26643
5	(advance*1 adj3 (plan*1 or planning or directive*)).tw,kf.	10168
6	hospice*.tw,kf.	13656
7	1 or 2 or 3 or 4 or 5 or 6	244157
8	exp Telemedicine/	35283
9	Telecommunications/ or exp Telemetry/ or Wireless Technology/ or exp Videoconferencing/	24389
10	Mobile Applications/ or exp Telephone/ or computers/ or microcomputers/ or computers, handheld/ or smartphone/ or minicomputers/ or User-Computer Interface/ or Computer assisted instruction/	145199
11	Internet-Based Intervention/	601
12	(((wearable or wireless) adj2 (technolog* or electronic*1 or device*1)) or (digital adj2 medicine) or (technolog* adj2 (remote or health)) or (remote adj2 care) or ((mobile or internet or electronic* or robot* or remote) adj2 (consultation* or application*1 or device*1))).tw,kf.	54138
13	(telecommunicat* or tele-communicat* or teleconferenc* or tele-conferenc* or app or apps or app-based or mobile-based or "Short Message Service*" or sms or textmessag* or text-messag* or texting or videoconferenc* or video-conferenc* or webconferenc* or web-conferenc* or webcast* or web-cast* or webinar* or web-application* or web-based-application*).tw,kf.	58430
14	(phone*1 or telephon* or smartphone* or smart-phone* or cellphone* or cell-phone* or mobilephone* or mobile-phone* or "personal digital assistant*" or palmpilot* or palm-pilot* or smarthome* or smart-home* or touchscreen* or "touch screen*" or "high tech*" or hightech*).tw,kf.	114251
15	(telemedicin* or tele-medicin* or telehealth* or tele-health* or telecare* or tele-care* or telecari* or tele-cari* or emedic* or e-medic* or ehealth* or e-health* or mhealth* or m-health* or ehomecare* or e-homecare* or e-home-care* or telenurs* or tele-nurs* or teletherap* or tele-therap* or telerehab* or tele-rehab* or erehab* or e-rehab* or teleconsultation* or tele-consultation* or videoconsultation* or video-consultation* or telemonitor* or tele-monitor*).tw,kf.	40192
16	("internet based intervention*" or "web based intervention*" or (digital adj2 intervention*)).tw,kf.	3256
17	8 or 9 or 10 or 11 or 12 or 13 or 14 or 15 or 16	371078
18	7 and 17	3477

19	exp Independent Living/ or Homebound Persons/	8411
20	exp Home Care Services/ or exp Community Health Nursing/	63980
21	((assisted or independent) adj2 living).tw,kf.	5740
22	"community dwelling".tw,kf.	25994
23	(homecare* or home-care*).tw,kf.	21267
24	(housebound or house-bound or homebound or home-bound or home or homes).tw,kf.	268132
25	((community adj2 (healthcare* or health-care* or nurs*)) or (community adj2 health adj2 service*) or "district nurs*").tw,kf.	19586
26	(remote or rural).tw,kf.	223956
27	19 or 20 or 21 or 22 or 23 or 24 or 25 or 26	547693
28	18 and 27	1041
29	limit 28 to yr="2000 -Current"	956
30	limit 29 to (chinese or danish or english or norwegian or portuguese or spanish or swedish)	924
